# *Jatropha curcas* L. Root Structure and Growth in Diverse Soils

**DOI:** 10.1155/2013/827295

**Published:** 2013-06-05

**Authors:** Ofelia Andrea Valdés-Rodríguez, Odilón Sánchez-Sánchez, Arturo Pérez-Vázquez, Joshua S. Caplan, Frédéric Danjon

**Affiliations:** ^1^Colegio de Postgraduados, Campus Veracruz 421, 91690 Veracruz, VER, Mexico; ^2^Centro de Investigaciones Tropicales, UV 91110 Xalapa, VER, Mexico; ^3^Department of Ecology, Evolution and Natural Resources, Rutgers, The State University of New Jersey, New Brunswick, NJ 08901, USA; ^4^INRA, UMR1202 BIOGECO, 33610 Cestas, France; ^5^Université de Bordeaux, UMR1202 BIOGECO, 33610 Cestas, France

## Abstract

Unlike most biofuel species, *Jatropha curcas* has promise for use in marginal lands, but it may serve an additional role by stabilizing soils. We evaluated the growth and structural responsiveness of young *J. curcas* plants to diverse soil conditions. Soils included a sand, a sandy-loam, and a clay-loam from eastern Mexico. Growth and structural parameters were analyzed for shoots and roots, although the focus was the plasticity of the primary root system architecture (the taproot and four lateral roots). The sandy soil reduced the growth of both shoot and root systems significantly more than sandy-loam or clay-loam soils; there was particularly high plasticity in root and shoot thickness, as well as shoot length. However, the architecture of the primary root system did not vary with soil type; the departure of the primary root system from an index of perfect symmetry was 14 ± 5% (mean ± standard deviation). Although *J. curcas* developed more extensively in the sandy-loam and clay-loam soils than in sandy soil, it maintained a consistent root to shoot ratio and root system architecture across all types of soil. This strong genetic determination would make the species useful for soil stabilization purposes, even while being cultivated primarily for seed oil.

## 1. Introduction

*Jatropha curcas* L. has received a great deal of attention for its potential as a biofuel crop due to the high oil content of its seeds and because it can grow in soils with low nutrient content or water availability and on thin or steeply sloping soils [1, 2]. *J. curcas* seedlings are known to have consistent root system architecture, with a prominent vertical taproot and four lateral roots branching at equal angles (90°). The structural characteristics of *J. curcas* roots may therefore provide soil resistance to water and wind erosion in some sites, while simultaneously providing seeds for biofuel production [3]. 

One problem in considering *J. curcas* for projects in degraded soils is that its response to varying soil conditions has not been quantitatively evaluated. There are indications that *J. curcas* may alter its growth patterns in response to suboptimal conditions. For example, it is capable of shedding its leaves during prolonged dry periods [4, 5]. However, Heller [6], who made qualitative observations of the species in the African continent, reported that *J. curcas* grows well even on gravelly, sandy, and saline soils. Although not based on quantitative data, his observations are still referenced frequently in efforts to promote *J. curcas* as a biofuel crop [1, 5]. In Mexico and Central America, where *J. curcas* is native, reports also state that it is normally found in marginal soils of low nutrient content [7, 8]. There are suggestions that the plant grows better in sandy and loamy (i.e., aerated) soils than in clayey soils [9, 10]. Clay soils are reportedly less suitable because they limit root system development, especially when they are saturated [10, 11]. However, Valdes et al. [12] found that *J. curcas* could be more productive in sandy-loam and clay-loam soils than in sandy soils.

While the basic patterns described in the literature on *J. curcas* may be accurate, the response of root structure to different soil conditions has never been evaluated directly, and aboveground responses are mainly based on observational studies. Knowledge of how *J. curcas* root system architecture varies across a range of soil types will facilitate an evaluation of its suitability for revegetation in soil conservation efforts, will be relevant for biofuel purposes, and may also help determine if both aims can be achieved simultaneously. The objective of this study was to quantitatively describe the shoot and root structural variation of *J. curcas* seedlings in three different soils that are characteristic of the Mexican tropics. 

## 2. Materials and Methods

### 2.1. Biological Material

Native Mexican seeds of *J. curcas* were collected in Papantla, in southeastern Mexico (20.2558° N, 97.2600° W, 77 masl) during August 2010. Seeds were selected from the middle of their weight distribution for sowing; average ± standard deviation (SD) measures were mass: 758 ± 97 mg, length: 8.4 ± 1.0 mm, width: 10.4 ± 0.50 mm, and thickness: 9.0 ± 0.5 mm. 

### 2.2. Soil Selection

Soils were selected based in their textural characteristics and because they represented prominent soils of the eastern Mexican tropics. The sandy soil was an arenosol, the sandy-loam was a regosol, while the clay-loam was a phaeozem; typologies were based on previous research performed in the region [13]. Sandy-loam and clay-loam soils were obtained from the premises of the Colegio de Postgraduados in Veracruz (19.1954° N, 96.3389° W), while sandy soil was obtained from a dune near the city of Veracruz (19.2093° N, 96.2597° W). The upper 50 cm of soil was collected and homogenized; one subsample (500 g) was taken from each soil type for physical and chemical analyses. Textural characterization was performed following Bouyoucos [14] and classified according to NRCS [15]; bulk density was estimated by the gravimetric method. Analysis of pH was conducted using an electronic potentiometer in a 1 : 1 slurry, organic matter content was determined by the Walkley-Black method, extractable phosphorus was determined following Olsen and Sommers [16], and exchangeable calcium and magnesium concentrations were determined using methods based on Diehl et al. [17], all adapted for Mexican soils [18]. 

### 2.3. Experimental Conditions

The experiment was conducted outdoors in Veracruz, Mexico (19.1988° N, 96.1522° W, 2 masl) and was carried out using a completely randomized design, with 15 replicates per soil type (clay-loam, sandy-loam, and sand; *n* = 45 plants). Seeds were sown in early September 2010 and were uprooted three months after germination (when they were in the juvenile life stage). The maximum, minimum, and average temperatures recorded at a local meteorological station (Skywatch Geos no. 11) during the period were 29.2, 19.4, and 23.7°C, respectively. The average relative humidity was 75.3%.

One seed was sown per pot, which consisted of a black polyethylene bag (40 cm diameter × 50 cm length) filled with the assigned soil. The soil in each bag was watered to field capacity daily to maintain near-constant moisture levels in all containers. Average irrigation provided per pot was approximately 310 mm (sand), 666 mm (sandy-loam), or 597 mm (clay-loam) in total through the experimental period. Pots with sandy soil received less water because of the lower water requirements of these plants. 

### 2.4. Aboveground Measurements

**Figure 1:**
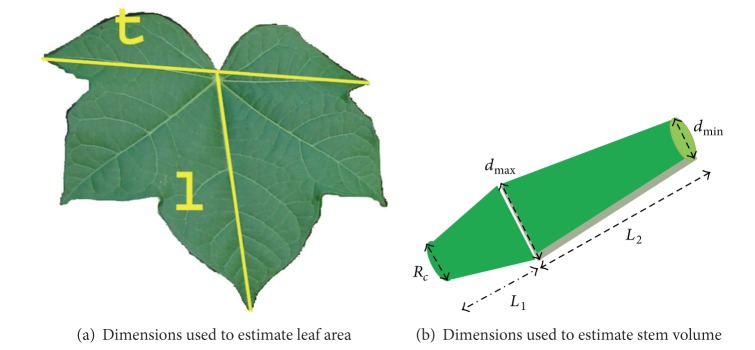
Leaf area and stem volume calculations.

At the conclusion of the experiment (three months after germination), we measured shoot length, leaf number, and diameter at the root collar. We also calculated the area of the largest leaf on each plant based on the model obtained by Liv et al. [19] for *J. curcas* (Figure 1(a)):

(1) $$\begin{array}{c} \text{Leaf Area}=0.84*{\left(t*l\right)}^{0.99}, \end{array}$$

where *t* = leaf cross-sectional length and *l* = leaf longitudinal length.

Stem volume (*V*) was calculated assuming that the stem was composed of two conical frustums: one extending from the root crown to the widest point on the stem and the other extending from the widest point to the attachment point of the most basal leaf (Figure 1(b)):

(2) V=π3∗L1∗[  (Rc2)2+(dmax⁡2)2+(Rc∗dmax⁡)]+π3∗L2∗[  (dmin⁡2)2+(dmax⁡2)2+(dmin⁡∗dmax⁡)],

where *R*_*c*_ is root collar diameter; *d*_max⁡_ is stem diameter at its widest point; *d*_min⁡_ is stem diameter at the attachment point of the most basal leaf; *L*_1_ is length from *R*_*c*_ to *d*_max⁡_; *L*_2_ is length from *d*_max⁡_ to *d*_min⁡_.

### 2.5. Uprooting

Plants were uprooted using methods that previous experience showed to be optimal for the various soil textures. Plants in sandy and sandy-loam soils were uprooted while the root zone was sprayed with water at low pressure. Plants in clay-loam soils were watered to 50% of the soil's saturation level and uprooted without the use of sprayed water. 

### 2.6. Identification and Digitization of the Root Structure

**Figure 2:**
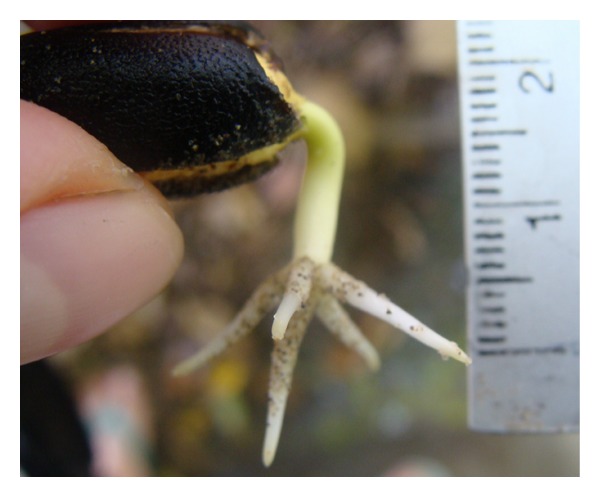
*Jatropha curcas *seedling 24 hr after germination. Note the radicle and four lateral roots which comprise its fundamental root structure.

**Figure 3:**
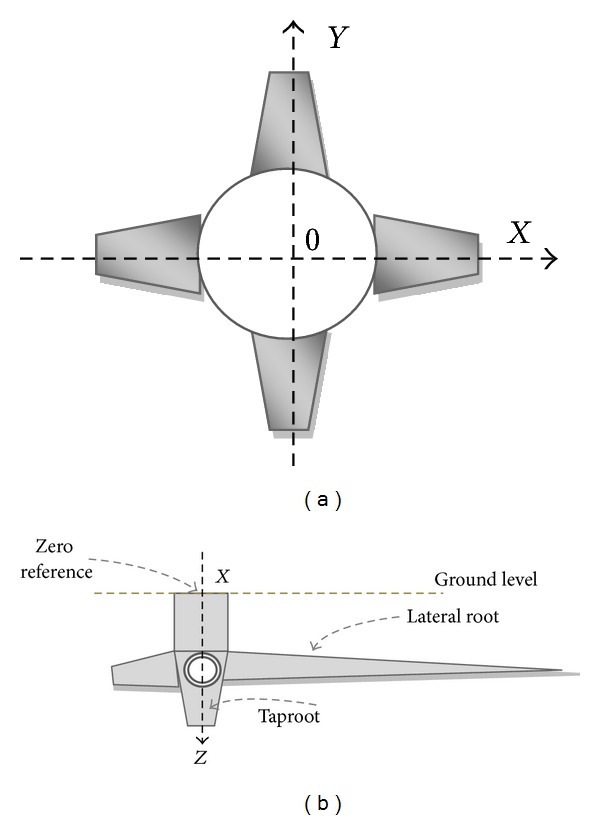
Coarse root structure. (a) Top view, (b) Lateral view.

The primary coarse root structure of *J. curcas *includes the taproot and four main lateral roots; these are all present within 24 hours of germination (Figure 2). The architecture of this set of five roots was encoded in 3D using methods adapted from Reubens et al. [3]. The taproot and the four primary lateral roots were encoded in terms of length (measuring tape, 1.0 mm precision), diameter (at bases and tips with a caliper, 0.01 mm precision), and orientation in the *X*, *Y*, and *Z* planes (at the bases and at 20 cm from their bases with a protractor, 1° precision). Secondary roots that emerged from any of the five primary roots and had a diameter thicker than 2.0 mm were also recorded. The soil level at the center of the stem base was considered the initial reference (0, 0, 0), while one of the four primary lateral roots was selected to define zero azimuth (Figure 3). Root segments ended at a branching point or where there was an abrupt change of growth direction. The above data were organized as Multi-scale Tree Graphs (MTGs), which are specialized databases for three-dimensional plant structure [20]. AMAPmod software version 2.2.30 [21] was used to derive architectural characteristics from the MTGs. Leaf, stem, and root dry masses were measured (analytical balance, 0.001 g precision) after oven drying at 70°C for 72 hr.

### 2.7. Modeling the Root Structure

**Figure 4:**
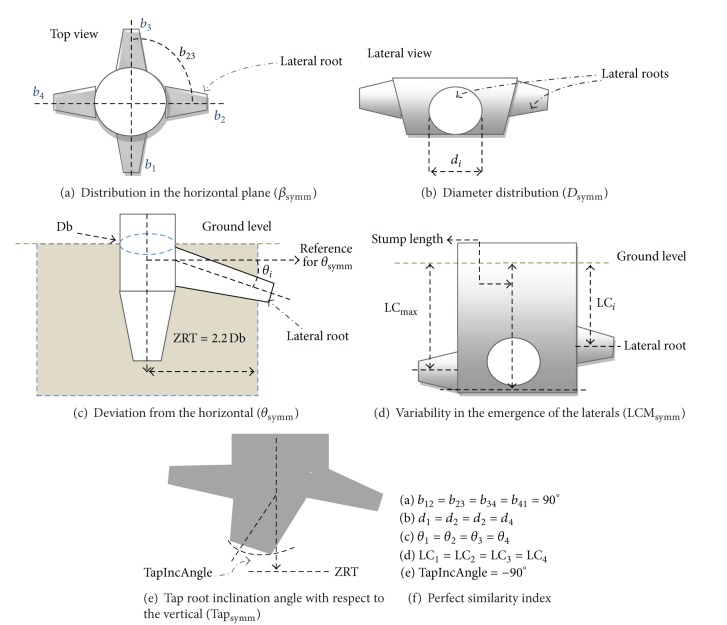
Parameters considered for calculating the similarity index and its component measurements.

In the idealized case, the four primary lateral roots of *J. curcas* would originate at the same vertical position along the stem, be symmetrically distributed in the horizontal plane, have the same diameters, and have the same inclinations. The consistency with which plants conformed to this idealized root structure was evaluated using a model that considers five estimators or indexes that range from zero to one, where zero is the perfect conformation to the idealized model and one represents maximal deviation from the model (modified from Reubens et al. [3], Figure 4). 

With respect to the horizontal plane, we considered the symmetry in the angular distribution of the four primary lateral roots (*β*_symm_):

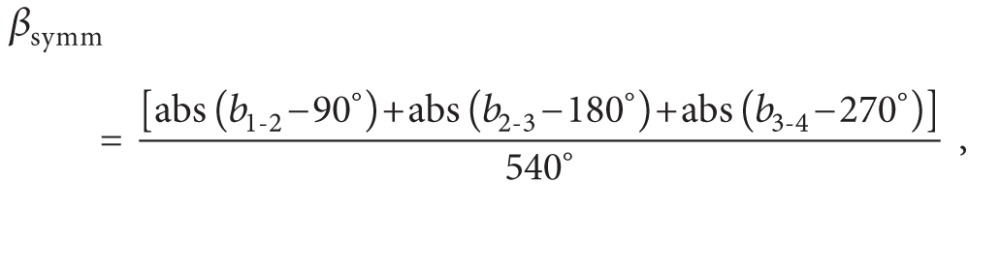
(3)
where *b*_*ij*_ is the horizontal angle between two neighboring primary lateral roots *i* and *j* (Figure 4(a)). *β*_symm_ = 0 if all the roots are distributed at 90° intervals and 1 if all the roots extend from a single point.

We also evaluated consistency in the basal diameter of the four primary lateral roots (*D*_symm_):

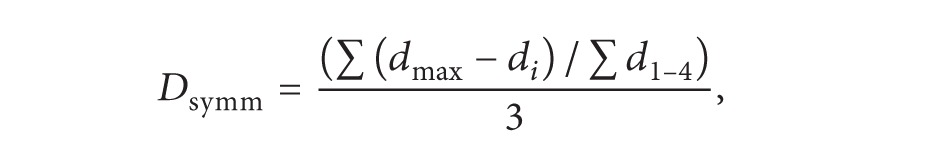
(4)
where *d*_*i*_ is the basal diameter of the *i*th primary lateral root (Figure 4(b)); ∑*d*_1–4_ is the sum of the four primary lateral root diameters; and *d*_max⁡_ is the maximum diameter of the four primary laterals. *D*_symm_ = 0 if all roots have the same diameter and 1 if there is only one lateral root.

In this study, instead of considering oblique roots, as in Reubens et al. [3], we considered the consistency in the angle of the four primary lateral roots below the horizontal surface (their inclinations, *θ*_*i*_), for the root within the ZRT (Zone of Rapid Taper, as defined by Danjon et al. [22]).

With respect to the vertical plane, we considered the symmetry in the angular deviation from the horizontal (*θ*_symm_):

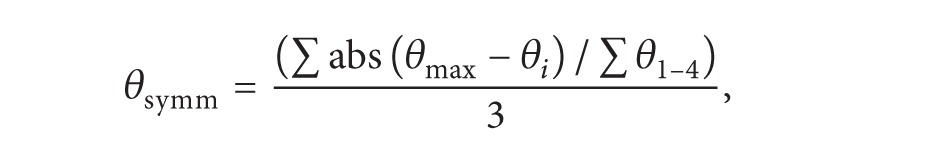
(5)
where *θ*_*i*_ is the angle of the *i* ^th^ primary lateral root below horizontal surface within the ZRT (Figure 4(c)); *θ*_max⁡_ is the maximum inclination of the four primary roots. *θ*_symm_ = 0 if all roots have the same inclination angle; 1 represents the highest difference between angles.

We also calculated the variability in the position of emergence of the four primary laterals from the taproot (LCM_symm_). To do this we considered the length from the root collar (at the level of the soil surface) to the branching point of each of the four primary lateral roots (LC_*i*_, Figure 4(d)):

(6) $$\begin{array}{c} {\text{LCM}}_{\text{symm}}=\;\left[\frac{\sum \left({\text{LC}}_{\max}-{\text{LC}}_{i}\right)}{3}\right]*\left(\text{Stump}\;\text{Length}\right), \end{array}$$

where LC_max⁡_ is the longest LC_*i*_, and Stump is the portion of the taproot from which the four main lateral roots branch [22] (Figure 4(d)). LCM_symm_ = 0 if all main laterals originate at the same point and 1 if roots originate from opposite extremes of the stump. Note that this definition of LCM_symm_ differs from that of Reubens et al. [3], insofar as they considered a departure from the fixed value of 2.5 cm for all seedlings in their study. As this value depends on the soil type and how deeply the seed was sown, we only evaluated the similarity of the length to root base collar (LC) from each lateral root.

The final similarity measurement we considered was the angle from which the taproot deviated from a vertical line (Tap_symm_):

(7) $$\begin{array}{c} {\text{Tap}}_{\text{symm}}=\frac{\left[\left(-90^{{}^{\circ}}\right)-\text{TapIncAngle}\right]}{90^{{}^{\circ}}},\;\;\;\;\;\;\;\; \end{array}$$

where TapIncAngle is the inclination angle between the taproot and the vertical at the level of the root stump (Figure 4(e)). Tap_symm_ = 0 if the taproot is vertically oriented and 1 if the taproot is horizontally oriented.

We computed a composite metric for the degree to which *J. curcas* plants adhered to the idealized model plant (SI):

(8) $$\begin{array}{c} \text{SI}=\frac{\left(\beta_{\text{symm}}+\;D_{\text{symm}}+\;\theta_{\text{symm}}+\;{\text{LCM}}_{\text{symm}}+{\text{Tap}}_{\text{symm}}\right)}{5}\;.\;\;\; \end{array}$$

SI = 0 for root systems perfectly matching the model and 1 for complete lack of adherence.

We used an index of phenotypic plasticity (PI) [23] to quantify the magnitude of the morphological response to varying soil types. For each variable, PI uses the mean response for individuals grown in each treatment to evaluate the greatest change displayed by the species among treatments:

(9) $$\begin{array}{c} \text{PI}=\frac{\left(\text{Maximum value}-\text{Minimum}\;\text{value}\right)}{\text{Maximum}\;\text{value}}. \end{array}$$

PI ranges from 0 to 1, with 1 representing the greatest possible plasticity.

### 2.8. Statistical Analysis

Differences in parameter means among soil types were statistically compared using one-way analysis of variance (ANOVA) in SigmaPlot 10.0. Tests of residual normality and equal variance were conducted. *Post hoc* comparisons were made for normally distributed parameters with a Tukey test, while non-normally distributed parameters were analyzed with Dunn's Method, all with a 95% confidence level.

## 3. Results

### 3.1. Soil Analysis

All substrates were found to be slightly alkaline. However, the sandy soil had very low organic matter content, being 2–4% of the amount in the other soils (Table 1). The sand also had 10–26% of the P, 23–44% of the Ca, and 29–53% of the Mg found in other soils (Table 1). 

### 3.2. Above- and Belowground Response to Soil Types

Plants grown in the sandy-loam and clay-loam soils had, on average, approximately twice the height and three times wider collar diameter than plants grown in sandy soil. Stem volumes, numbers of leaves, and leaf areas were more than five times greater for plants grown in these soils than for plants grown in sandy soil (Table 2). All plants grown in sandy soil survived, but 62% were completely defoliated by the conclusion of the experiment; none of those grown in sandy-loam or clay-loam soils lost all leaves. Stem slenderness ratio (height over root collar diameter) did not differ among soils, indicating that the seedlings were not plastic in this trait. 

Root diameters and volumes for the five primary roots in *J. curcas* differed significantly among soil types (*P* < 0.001). Secondary root growth (thickening) was lower for plants in sandy soil than those in sandy-loam or clay-loam soils. Roots in sandy- and clay-loams had similar basal and apical diameters. They also had a greater number of branches thicker than 2.0 mm and larger volumes than those in sandy soil. All taproots in sandy-loam and clay-loam soils developed secondary roots thicker than 2.0 mm, whereas only 13% of the taproots in sandy soil developed such roots. However, root lengths did not differ significantly among treatments (*P* > 0.05). 

**Figure 5:**
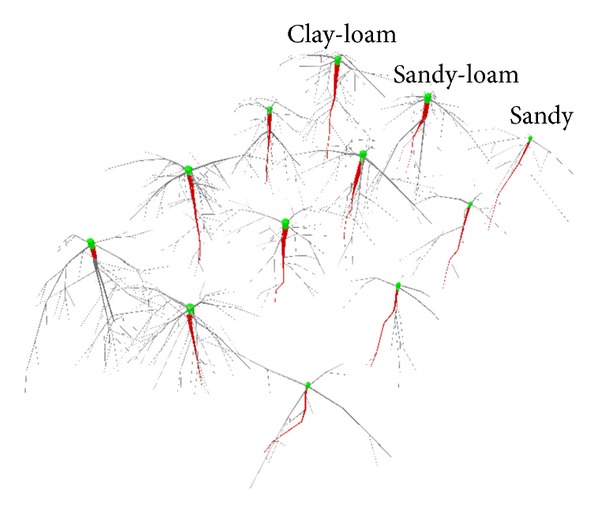
Digitized *Jatropha curcas* root systems grown in three different soils.

**Figure 6:**
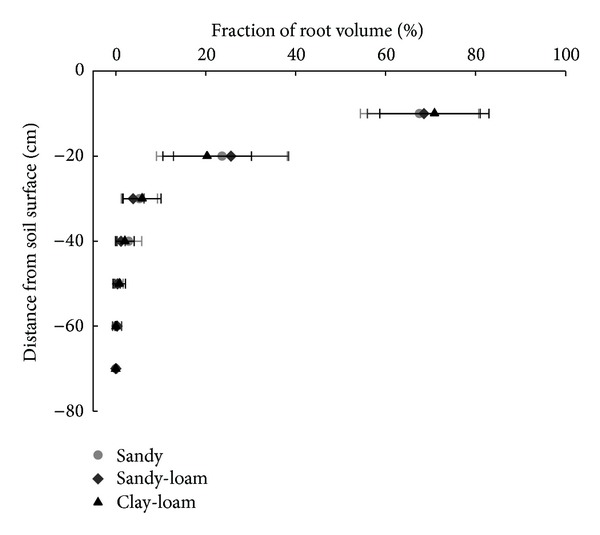
Root volume distribution by depth in sandy, sandy-loam and clay-loam soils.

Stem mass, leaf mass, and root system mass were lower at the conclusion of the three-month growing period in sandy soil than in clay-loam or sandy-loam soils (Table 2, Figure 5). Despite these differences, allocation of biomass was greater to stems than to roots in all three soil types (Table 3). Within root systems, the greatest proportion of biomass and volume was allocated to taproots (Table 4). The uppermost 10 cm of soil contained the majority of root volume (Figure 6).

### 3.3. Root Structure, Similarity Indices, and Plasticity to Soil

Root system symmetry index scores were typically 0.146 ± 0.05 (mean ± SD); mean SI did not statistically differ among soil types (Table 4). Of the main parameters defining *J. curcas* root structure, taproot inclination, and primary lateral root distribution (β) had the lowest plasticity index scores (0.05 and 0.06, respectively). Biomass allocation to roots and the inclination angles (*θ*), length, and apical diameters of the five primary coarse roots also showed low plasticity (PI < 0.31). However, the number of secondary lateral roots thicker than 2 mm, as well as root mass, was highly plastic (*P* > 0.63) (Table 4).

## 4. Discussion

### 4.1. Growth and Mass Distribution

*J. curcas* had a significant growth response to soil conditions. In sandy soil, it displayed characteristics typical of plants grown in arid conditions, including reduction of leaf area and defoliation. These responses reduce transpirational surface area and are common adaptations of species with photosynthetic stems, such as *J. curcas* [24]. Additionally, the low nutrient availability of the sandy soil strongly reduced stem and leaf growth [25, 26]. Higher biomass allocation to stems over leaves and roots, regardless of soil type, indicates that this ratio is strongly genetically determined. This lack of plasticity may be adaptive because stem tissue is used for water storage by seedlings of *J. curcas*, allowing them to survive during dry periods [5]. Another pattern that remained consistent across soil types was that the largest fraction of the mass was allocated to roots in the uppermost 10 cm of the surface (Figure 4) and to the taproot (73% in sand, 87% in sandy-loam, and 76% in clay-loam) (Table 3). The root architecture of one-month-old seedlings grown in sandy soil was previously described by Reubens et al. [3]; they had 50% of their root volume allocated to the taproot. Taken together, these results suggest that there is an increase in the mass and volume of taproot as compared to lateral roots over time. Enlarged taproots, in combination with consistently shallow lateral roots, indicate that seedlings search simultaneously for resources in deep and shallow soil. 

Although the clay-loam soil used in this experiment had the highest nutrient content, there were no differences in growth parameters or biomass compared to the sandy-loam, which had a lower nutrient content (Tables 2 and 3). This result is contrary to that of Patolia et al. [27], who reported greater biomass production under elevated nutriment conditions. In this study, it is likely that plant roots were more easily able to obtain nutrients from the sandy-loam than from the clay-loam because of the high soil aeration requirements of *J. curcas*. It is also possible that nutrient levels in our sandy-loam soil were near the ideal levels to which this species is adapted at this stage of growth [28].

Stem growth rates of 4.1 mm day^−1^ recorded in sandy-loam and clay-loam soils were similar to growth rates measured by Jimu et al. [29] in clay soils and under similar temperatures. However, the low aboveground development in sandy soil found in this study (2.3 mm day^−1^) contrasts with claims that *J. curcas* can grow well under semiarid conditions and sandy soils [6, 9]. Higher stem and leaf growth rates in sandy soil have been reported before [30] but under periodic water irrigation with amendments of N, P, K, Ca, and Mg. Low levels of N and P in our sandy soil probably contributed to leaf loss and slow stem growth rates [31]. Maintenance of root length in sandy soil, despite extreme reductions in root mass, indicates that plants retain a capacity for soil exploration under limiting nutrient conditions. Similar patterns were reported by Achten et al. [30] under extreme drought stress. This strategy may serve to improve foraging outcomes for soil resources. Each of the five primary roots in *J. curcas* took on a strongly herring-bone branching structure. This structure is highly efficient in soil exploration [32] and is indicative of *J. curcas*' adaptation to well-drained and nutrient-poor soils [33, 34].

### 4.2. Root Structure and Plasticity to Soil Type

As observed previously by Reubens et al. [3], we found similar symmetry index values among soils. Primary lateral root and taproot inclination angles were also similar among the three soils, suggesting that the arrangement and structure of *J. curcas* root systems are strongly determined by genetics and only weakly affected by environmental conditions, such as the soil textures used in this experiment. Having prominent lateral roots with a symmetrical radial distribution and consistent diameters provides balanced anchorage to *J. curcas* plants; this root structure can tolerate forces originating from varying directions and maintain stability. Low plasticity in stem allocation, root allocation, and root structure (Tables 3 and 4) indicates that these characteristics are also strongly determined by genetics and are minimally influenced by soil conditions. Maintenance of higher mass in stems than in roots, independent of the soil condition, may also indicate that *J. curcas* is a species that evolved to store resources in the stem and thereby avoid physiological stress in extreme environmental conditions [23]. Positioning lateral roots near the soil surface is a characteristic of plants adapted to arid climates [24]. Therefore, this species could be established in sites with limited nutrient and water resources, although growth rates and seed production under these circumstances could be extremely low.

The fact that the primary root system structure of *J. curcas* (a long, thick taproot with four, nearly perpendicular lateral roots) was not plastic in response to soil type indicates that its large lateral roots are able to stabilize superficial soils, while its large taproot can provide reinforcement across planes of weakness, for example, along the flanks of potential slope failures [22, 35]. Therefore, this plant will reliably reinforce soils in which it is planted by increasing the shear and tensile strength of the rooting zone [36]. Additionally, *J. curcas* has been shown to raise the macroaggregate stability and organic matter content of the soils in which it grows [37], ensuring that precipitation infiltrates rather than runs off and that a minimal amount of soil erodes.

## 5. Conclusions

*J. curcas* seedlings developed well in both sandy-loam and clay-loam soils. In sandy soil, its growth was reduced significantly, though plants were still able to survive and maintain a favorable root-shoot relationship. These characteristics would allow the plant to survive under a wide variety of soil conditions, making it well suited for preventing soil erosion. Although its growth, seed production, and performance for erosion control could be lower in poor soils, *J. curcas *cultivation programs could not only serve as a source of income generation, but could also improve the quality of soils in the long run. 

## Figures and Tables

**Table 1:** Soil characteristics for the three soil types in which *J. curcas* seedlings were grown for three months.

Soil type	Texture (%)			pH	Bulk density (g cm^−3^)	Organic matter (g kg^−1^)	P (g kg^−1^)	Ca (mmol kg^−1^)	Mg (mmol kg^−1^)
	Sand	Silt	Clay						
Sand	96.0	2.5	1.5	7.81	1.56	1.68	0.01	77.17	154.35
Sandy-loam	66.0	21.0	13.0	7.26	1.47	39.00	0.05	175.40	294.66
Clay-loam	30.0	35.0	35.0	7.43	1.26	72.62	0.12	329.74	519.17

**Table 2:** Aboveground parameters in *J. curcas *seedlings grown in three different soils.

Soil type	Stem length (mm)	Root collar diameter (mm)	Stem slenderness (cm cm^ 1^)	Stem volume (cm^3^)	Number of leaves	Leaf area (cm^2^)
Sand	209.4 ± 26.6^b^	12.1 ± 1.6^b^	17.5 ± 2.7^a^	21.71 ± 8.9^b^	0.5 ± 0.8^b^	29.5 ± 0.2^b^
Sandy-loam	380.2 ± 88.7^a^	23.4 ± 3.3^a^	15.9 ± 2.9^a^	118.78 ± 45.7^a^	7.0 ± 2.6^a^	223.0 ± 5.4^a^
Clay-loam	361.6 ± 72.8^a^	23.1 ± 3.0^a^	15.6 ± 1.7^a^	114.11 ± 46.8^a^	6.8 ± 2.8^a^	178.0 ± 54.9^a^

^a,b^Means within a column which do not share the same letter are significantly different (*P* < 0.05).

**Table 3:** Average ± SD dry matter allocation in *J. curcas curcas* grown in three different soil types.

Soil type	Total biomass (g)	Stem, total^−1^	Leaves, total^−1^	Root, total^−1^
Sand	3.17 ± 1.24^b^	0.77 ± 0.10^a^	0.03 ± 0.04^b^	0.20 ± 0.07^a^
Sandy-loam	29.59 ± 9.81^a^	0.63 ± 0.06^a^	0.19 ± 0.06^a^	0.18 ± 0.03^a^
Clay-loam	30.01 ± 11.01^a^	0.66 ± 0.10^a^	0.17 ± 0.04^ab^	0.17 ± 0.04^a^

^a,b^Means within a column which do not share the same letter are significantly different (*P* < 0.05).

**Table 4:** Average ± SD below-ground parameters in *J. curcas* seedlings grown in three different soils.

Parameter	Units	Sand	Sandy-loam	Clay-loam	PI
Root length	cm				
Total		115.9 ± 17.0^b^	132.0 ± 18.2^ab^	144.6 ± 38.6^a^	0.20
Taproot		36.1 ± 85.1^a^	39.7 ± 95.9^a^	41.1 ± 72.1^a^	0.12
Four main laterals		27.7 ± 8.5^a^	32.3 ± 10.1^a^	37.5 ± 14.6^a^	0.26
Basal diameter	mm				
Taproot		8.2 ± 1.3^b^	20.1 ± 4.4^a^	18.4 ± 3.2^a^	0.59
Four main laterals		2.9 ± 0.4^b^	4.8 ± 1.9^a^	5.2 ± 1.3^a^	0.44
Apex diameter	mm				
Taproot		0.70 ± 0.25^a^	0.63 ± 0.17^a^	0.70 ± 0.15^a^	0.10
Four main laterals		0.41 ± 0.11^b^	0.53 ± 0.09^ab^	0.59 ± 0.19^a^	0.31
Number of roots > 2.0 mm thick		5.13 ± 0.35^b^	12.09 ± 5.85^a^	13.89 ± 5.84^a^	0.63
Root mass					
Total	g	0.61 ± 0.20^b^	5.33 ± 1.74^a^	5.34 ± 3.02^a^	0.89
Taproot	%	74.40 ± 9.85^b^	85.48 ± 6.97^a^	75.78 ± 7.05^b^	
Coarse root structure					
TapIncAng	deg	−89.36 ± 4.48^a^	−85.21 ± 3.72^a^	−88.67 ± 6.12^a^	0.05
*θ*	deg	−20.71 ± 4.79^a^	−18.59 ± 3.05^a^	−17.94 ± 3.31^a^	0.13
LCM	cm	1.07 ± 0.41^a^	1.36 ± 0.50^a^	1.0 ± 0.38^a^	0.26
*β*	deg	89.3 ± 4.40^a^	94.7 ± 3.7^a^	91.3 ± 6.1^a^	0.06
Similarity indexes					
*β*_symm_		0.02 ± 0.02^a^	0.04 ± 0.03^a^	0.03 ± 0.03^a^	
*D*_symm_		0.24 ± 0.10^a^	0.29 ± 0.09^a^	0.25 ± 0.14^a^	
*θ*_symm_		0.27 ± 0.10^a^	0.34 ± 0.11^a^	0.35 ± 0.12^a^	
LCM_symm_		0.12 ± 0.06^a^	0.16 ± 0.13^a^	0.11 ± 0.07^a^	
Tap_Symm_		0.04 ± 0.04^a^	0.04 ± 0.03^a^	0.04 ± 0.03^a^	
SI		0.13 ± 0.05^a^	0.15 ± 0.07^a^	0.16 ± 0.03^a^	

^a,b^Means within a column which do not share the same letter are significantly different (*P*< 0.05).
